# Supplementary material for: Research on the impact of digital financial inclusion and environmental regulation on industrial structure upgrading-based on spatial Durbin model analysis
Source: PLoS One. 2024 Nov 21;19(11):e0310720. doi: 10.1371/journal.pone.0310720 (PMC11581305; doi:10.1371/journal.pone.0310720)
Supplement: Attachment — Submitted filename: 01-Answer letter.docx [file pone.0310720.s001.docx]

Prof. Gui-mei Zhang PhD

School of Economics and Management, Sanming University, Sanming 353000, China.

April 06, 2024

Dear Professor Habil.,

Glad to receive your E-mail for informing me that our paper entitled **“Digital Finance Inclusive, Environmental Regulation, and Industrial Structure Upgrade: A Spatial Durbin Model Analysis Based on Provincial Data in China” (No:** [PONE-D-24-02477]**)** needs major revisions and clarification before considering for publishing in **PLOS ONE**. Thanks for your help and the helpful comments and suggestions from the reviewers. The manuscript has been thoroughly revised according to the reviewers’ comments, and the answers to the comments are listed below:

In response to the latest editorial feedback, we have made the following revisions:

1. We updated the “Funding Information”.
2. We uploaded a minimal data as “Supporting Information”.
3. We have changed the article title to English.
4. We updated the source of map images used and provide the relevant link:

[http://bzdt.ch.mnr.gov.cn/editmap.html?mapId="4028b0625501ad13015501ad2bfc0002"&baseMapId="4](http://bzdt.ch.mnr.gov.cn/editmap.html?mapId=%224028b0625501ad13015501ad2bfc0002%22&baseMapId=%224)"

Thus, we made them consistent.

**Reviewer #1:**

**Comment 1:** The authors from the results section never mention any implication for academics, stakeholders and practitioners. I suggest them to better address the results, the implications and the relevance of the study.

**Author’s response:** Thank the reviewer for the comment. We have added the relevant content in the revised the manuscript **(See Lines 955-966)**.

**Comment 2:** The attention to the language is not capillary. Some sentences are fragmented, and others are not properly written. I suggest the authors to make a professional English proofreading.

**Author’s response:** Thank the reviewer for the comment. We have revised the whole manuscript carefully and tried to avoid any grammar or syntax error. In addition, we had invited native English speaker to review this manuscript, and avoided any mistakes of tense, grammar and syntax in the new version. We believe that the language is now acceptable for the review process.

**Comment 3:** To address potential issues in the methodology related to spatial econometric modeling, three specific tests can be conducted. These tests aim to validate the model's assumptions, check for robustness, and ensure the reliability of the findings:

Test 1: Test for Spatial Autocorrelation

Purpose: To detect the presence of spatial autocorrelation in the residuals of the econometric model, which would indicate that the model has not fully captured spatial dependencies among regions.

Use Moran's I test on the residuals of the regression model. Moran's I is a measure of spatial autocorrelation and can indicate whether the residuals are randomly distributed across space or if there is a pattern that the model has failed to capture.

If significant spatial autocorrelation is found, it suggests that the model might need to incorporate additional spatial terms or use a different spatial weighting matrix.

**Author’s response:** Thank the reviewer for the comment. To support our methodology, we have referenced numerous papers that have utilized the Moran I test, published in journals such as **PLOS ONE**. Additionally, we have conducted the Moran I test as part of our analysis. **(See Line 636 and Table 3 Global spatial autocorrelation test result values)**.

**Comment 4:** Test 2: Robustness Checks Using Alternative Spatial Weight Matrices

Purpose: To assess how sensitive the model's results are to the choice of the spatial weighting matrix. This is crucial since the spatial weight matrix defines the structure of spatial interactions and dependencies among the units of analysis.

Re-estimate the model using alternative definitions of the spatial weighting matrix. For example, besides the economic geography matrix and the square of the road distance, consider matrices based on physical distance, shared borders, or even non-spatial relationships like economic similarity or historical trade links.

Compare the results across different matrices to check for consistency in the sign, magnitude, and significance of estimated parameters. Significant variations could indicate that the model's findings are highly sensitive to the choice of spatial matrix, necessitating further investigation.

**Author’s response:** Thank the reviewer for the comment. I have added the use of provincial distance matrices as a robustness test. **(See Lines 848)**. Other matrices have not been explored in this paper and remain one of the outstanding issues to be addressed in the article.

**Comment 5:** Test 3: Cross-validation for Model Specification

Purpose: To validate the model specification and ensure that the chosen variables and their interactions robustly explain the industrial structure upgrading without overfitting the data.

Implement a cross-validation technique, such as k-fold cross-validation, where the dataset is divided into k subsets. The model is trained on k-1 subsets and tested on the remaining subset, and this process is repeated k times with each subset used once as the test set.

Evaluate the model's performance across different folds using metrics such as R-squared, mean squared error (MSE), or mean absolute error (MAE) for both training and validation sets. Consistent performance across folds suggests that the model is robust and not overfitting.

**Author’s response:** Thank the reviewer for the comment. We have added the Cross-validation for Model Specification (ER* DFI). **(See Lines 852, Table 10 Robustness test)**.

**Reviewer #2:**

**Comment 1:** The authors have not adequately delineated the criteria for sample selection in their methodology, leaving a significant gap in understanding the research framework.

**Author’s response:** Thanks so much for the comments. I have fully articulated the criteria for sample selection in the methodology of the revised draft. **(See Lines 614-623)**.

**Comment 2:** The authors have not sufficiently explained the study's implications or its innovative aspects, which limits the reader's understanding of its importance and original contribution to the finance literature.

**Author’s response:** Thank the reviewer for the comment. We have revised the research significance and innovation of the article. **(See Lines 94-102)**. We also add the conclusion of the article. **(See Lines 919-937)**.

**Comment 3:** - The authors have not articulated the limitations of their study, an omission that restricts the scope of its interpretability and applicability in broader contexts..

**Author’s response:** Thank the reviewer for the comment. We have added the limitations of the study. **(See Lines 938-954)**

**Comment 4:** The study's contribution to the existing body of knowledge appears limited as it explores the relationship between environmental regulation, digital financial inclusion (DFI), and the upgrading of industrial structures—a topic already well-covered in the literature. The empirical findings, derived from panel data analysis across 30 Chinese provinces and employing the spatial Durbin model, reaffirm the established understanding that both DFI and environmental regulation can significantly influence industrial structure upgrades locally and in neighboring regions. Furthermore, the identification of a single-threshold effect characterized by an inverted 'U' relationship, along with the noted regional heterogeneity in the effects of DFI and environmental regulation, aligns with prior studies. These findings, while valuable, do not introduce novel insights into the dynamics between DFI, environmental regulation, and industrial upgrading but rather reinforce the recognized importance of these factors in promoting sustainable development. The study's emphasis on the need for a balanced approach to DFI and environmental regulation, to avoid mission drift and ensure effective industrial upgrading, underscores existing recommendations rather than offering new theoretical or methodological advancements.

**Author’s response:** Thank the reviewer for the comment. There is innovation in our article. In particular, the mechanism of action part of the article is strictly based on the theoretical analysis of spatial direct and indirect effects. In addition, the article analyzes the impact of the interaction terms of DFI and environmental regulation on industrial structure upgrading.

**Reviewer #3:**

**Comment 1:** The paper's contribution to the existing literature on environmental regulation and digital financial inclusion (DFI) in the context of industrial upgrading is limited, as it reiterates already well-established findings within the field. The methodology and results, while thorough, do not introduce new theoretical perspectives or empirical evidence that advances the understanding of these topics. The analysis aligns closely with previous studies, and the conclusions are consistent with established knowledge, resulting in a lack of innovative insights.

**Author’s response:** Thank the reviewer for the comment. There is innovation in our article. In particular, the mechanism of action part of the article is strictly based on the theoretical analysis of spatial direct and indirect effects. In addition, the article analyzes the impact of the interaction terms of DFI and environmental regulation on industrial structure upgrading.

**Comment 2:** Introduction. The introduction lacks a clear and concise research objective that directly states the study's primary aim, creating ambiguity regarding the specific problem the research intends to address within the broader context of digital finance inclusion, environmental regulation, and industrial structure upgrading.

**Author’s response:** Thank the reviewer for the comment. We have rewritten the introduction section. **(See Lines47-93)**

**Comment 3:** I recommend that the Author(s) restructure the literature review to better align with the paper's research objectives and to enhance its contribution to the field. This could involve a more focused examination of previous studies that directly relate to digital finance inclusion (DFI), environmental regulation, and industrial upgrading, particularly those employing spatial econometric models. Emphasizing research from highly ranked journals would strengthen the review’s foundation.

**Author’s response:** Thank the reviewer for the comment. We have moderately adjusted the literature review by adding relevant literature. **(See Lines108-114;157-158;162-164;166-178;200-203;214-216)**However, there are still fewer studies directly related to digital financial inclusion (DFI), environmental regulation and industrial upgrading in the first place.

**Comment 4:** The Author(s) should also critically assess the methodologies and findings of these studies, identifying gaps your research aims to address. Moreover, a discussion on the variable impacts of DFI and environmental regulation across different regions, especially within the context of China, would add depth.

**Author’s response:** Thank the reviewer for the comment. We have added reviews of the literature.

**Comment 5:** Concluding the section with a clear articulation of how the current study extends or fills gaps in existing literature could clarify its novel contribution. A conclusion paragraph in the literature review section is redundant.

**Author’s response:** Thank the reviewer for the comment. We have revised the literature innovations. **(See Lines 217-225)**.And ,we deleted some of them.

**Comment 6:** Sample and Methodology. The sample must be specified. This information is only provided in the abstract.

**Author’s response:** Thank the reviewer for the comment. W We have supplemented the sample description section. **(See Lines 614-623)**.

**Comment 7:** To enhance the robustness of the findings and address potential concerns regarding the spatial econometric model's accuracy and applicability, the Author(s) could consider the following tests: Lagrange Multiplier (LM) Tests for Spatial Dependence: Conduct Lagrange Multiplier tests for both spatial lag and spatial error models to detect any hidden spatial dependence not captured by the initial model. This can help justify the choice of a spatial econometric model over traditional regression models.

**Author’s response:** Thank the reviewer for the comment. We have already done the relevant tests in the article. **(See Lines 688 Table 4 Results of the spatial Durbin model applicability test)**.

**Comment 8:**Cross-Validation with Non-Spatial Models: Compare the results from the spatial econometric model with those obtained from non-spatial econometric models, such as OLS or fixed-effects models, with and without spatially lagged independent variables. This comparison can validate the significance of incorporating spatial effects into the.

**Author’s response:** The choice of the spatial Durbin model is the most appropriate due to the tests that have been done earlier in this paper. So both spatial lag and spatial error models are not listed in the writing of this paper.

**Comment 9:**Results. The presentation of the results is unclear, and the discussion section fails to convey the study's implications and significance adequately.

**Author’s response:** Thank the reviewer for the comment. We have revised the conclusion section of the article. **(See Lines 854-872)**. We've also added a revelation section to the article. **(See Lines 919-937)**.

**Comment 10:** Conclusion and Implications. In the conclusion section, the Author(s) omits a discussion of the study's limitations and implications, which is essential for providing a comprehensive understanding of the research's scope, applicability, and potential impact on the field.

**Author’s response:** Thank the reviewer for the comment. We have added a limitations section to the article. **(See Lines 939-954)**.

We have reviewed point by point in the new revised version, marked by red character, according to your comments. I hope the above answers and revisions are satisfactory, and the paper will be acceptable for publication in ***PLOS ONE***.

Best wishes,

Dr. Gui-mei Zhang
