# Supplementary material for: Research on the impact of digital financial inclusion and environmental regulation on industrial structure upgrading-based on spatial Durbin model analysis
Source: PLoS One. 2024 Nov 21;19(11):e0310720. doi: 10.1371/journal.pone.0310720 (PMC11581305; doi:10.1371/journal.pone.0310720)
Supplement: Attachment — Submitted filename: REV 2 PLOS.docx [file pone.0310720.s002.docx]

REV 2 PLOS

I appreciate your diligent efforts made in revising your manuscript. I am pleased to observe that all of my previous comments and suggestions have been thoroughly addressed. The comprehensive manner in which you have incorporated these changes is commendable, and it is evident that the quality and clarity of the manuscript have been significantly enhanced as a result.

The revised version of your paper now aligns well with the high standards expected by the journal, reflecting both the robustness of your research and the depth of your analysis. Your commitment to improving the manuscript is truly admirable.

Congratulation!
