# Supplementary material for: Research on the impact of digital financial inclusion and environmental regulation on industrial structure upgrading-based on spatial Durbin model analysis
Source: PLoS One. 2024 Nov 21;19(11):e0310720. doi: 10.1371/journal.pone.0310720 (PMC11581305; doi:10.1371/journal.pone.0310720)
Supplement: Attachment — Submitted filename: Response to Reviewers.docx [file pone.0310720.s003.docx]

Prof. Gui-mei Zhang PhD

School of Economics and Management, Sanming University, Sanming 353000, China.

August 01, 2024

Dear Professor Habil.,

Glad to receive your E-mail for informing me that our paper entitled **“Digital Finance Inclusive, Environmental Regulation, and Industrial Structure Upgrade: A Spatial Durbin Model Analysis Based on Provincial Data in China” (No:** [PONE-D-24-02477]**)** needs major revisions and clarification before considering for publishing in **PLOS ONE**. Thanks for your help and the helpful comments and suggestions from the reviewers. The manuscript has been thoroughly revised according to the reviewers’ comments, and the answers to the comments are listed below:

**Additional Editor Comments:**

The manuscript still requires further revision towards the introductory section, as well as the methodological approach. As well, the quality of the English language should be improved.

**Author’s response:** Thank the editor for the comment. We have revised the introduction and methodology sections as required, and we have also thoroughly checked the entire text. **(See Lines 45-54;76-85;94-96;557-587)**

**Reviewer #1:**

**Comment 1:** The introduction should provide a clearer and more concise research objective. This will help eliminate any ambiguity regarding the specific problem the study intends to address within the context of digital finance inclusion, environmental regulation, and industrial structure upgrading.

**Author’s response:** Thank the reviewer for the comment. We have revised the introduction and rewritten some parts, making the issues clearer. **(See Lines 45-54;76-85;94-96)**.

**Comment 2:** Additionally, the methodology section needs a detailed explanation of the criteria for sample selection. This will strengthen the research framework and ensure a better understanding of the study's context.

**Author’s response:** Thank the reviewer for the comment. We have provided additional explanations and made the relevant revisions. **(See Lines 557-574;579-581)**

**Comment 3:** Furthermore, the implications and innovative aspects of the study should be elaborated upon to highlight its importance and original contributions to the finance literature.

**Author’s response:** Thank the reviewer for the comment. We have summarized the innovative points of the article. **(See Lines 86-96)**

**Comment 4:** This includes discussing the study's limitations, as this is crucial for understanding the scope and applicability of the research in broader contexts. Lastly, ensure that the presentation of results and the discussion sections clearly convey the study's significance and implications, enhancing the overall comprehensibility of the manuscript

**Author’s response:** Thank the reviewer for the comment. We have supplemented and revised this section accordingly. **(See Lines 885-899)**. We have also rewritten the conclusion. **(See Lines 806-821).**

**Comment 5:**The language throughout the manuscript needs careful proofreading to address fragmented sentences and improve overall readability. Professional English proofreading is recommended to avoid any grammatical or syntactical errors.

**Author’s response:** Thank the reviewer for the comment. We have revised the whole manuscript carefully and tried to avoid any grammar or syntax error. In addition, we had invited native English speaker to review this manuscript, and avoided any mistakes of tense, grammar and syntax in the new version. We believe that the language is now acceptable for the review process.

**Comment 6:** Additionally, the literature review should be better aligned with the paper's research objectives, including a focused examination of previous studies related to digital finance inclusion, environmental regulation, and industrial upgrading, especially those employing spatial econometric models. Emphasizing research from highly ranked journals will strengthen the foundation of the literature review.

**Author’s response:** Thank the reviewer for the comment. We have revised parts of the literature review and included articles with a high impact on spatial econometrics related to the article's theme. **(See Lines 111-114;159-168;185-190;202-205)**.

**Comment 7:** Finally, a more critical assessment of the methodologies and findings of these studies should be included, identifying gaps that your research aims to address and articulating how the current study extends or fills these gaps, thereby clarifying its novel contributions.

**Author’s response:** We have further verified the article. The empirical results effectively support the hypotheses and include robust regression analyses

**Reviewer #2:**

**Comment 1:** I appreciate your diligent efforts made in revising your manuscript. I am pleased to observe that all of my previous comments and suggestions have been thoroughly addressed. The comprehensive manner in which you have incorporated these changes is commendable, and it is evident that the quality and clarity of the manuscript have been significantly enhanced as a result. The revised version of your paper now aligns well with the high standards expected by the journal, reflecting both the robustness of your research and the depth of your analysis. Your commitment to improving the manuscript is truly admirable.
Congratulation!

**Author’s response:** Thanks so much for the comments. Thank you for your guidance.

**Reviewer #3:**

**Comment 1:** 1) The title of the manuscript could be streamlined and made more explanatory. According to Ecarnot et al. (2015), “The title should contain keywords to reflect the main issues in your article. It should also awaken the potential reader’s interest and incite the desire to read your work in full”.

**Author’s response:** Thank the reviewer for the comment. We have revised the title according to your request. **(See Lines1-3)**

**Comment 2:** The abstract could be improved; the current version does not highlight the study's relevance, novelty, and contribution. To enhance its clarity and impact, please following these suggestions:

- Conciseness and clarity: Simplify sentences to enhance readability and ensure that each

sentence conveys a clear, single idea.

- Logical flow: Ensure that the abstract follows a logical progression from the introduction of the problem through methodology to key findings and implications.

- Focus on key results: Emphasize the most significant findings and their implications rather than providing extensive details on regional differences or specific effects.

**Author’s response:** Thank the reviewer for the comment. We have rewritten the abstract section. **(See Lines23-40)**

**Comment 3:** The keywords should be refined in order to accurately represent the topic developed in your study and ensure coherence with the JEL classification.

**Author’s response:** Thank the reviewer for the comment. We have made the necessary revisions as requested. **(See Lines41-42)**

**Comment 4:** It is essential to conduct a thorough proofreading of the manuscript. This will help refine the constructs and enhance the overall quality of the writing.

**Author’s response:** Thank the reviewer for the comment. We have revised the whole manuscript carefully and tried to avoid any grammar or syntax error. In addition, we had invited native English speaker to review this manuscript, and avoided any mistakes of tense, grammar and syntax in the new version. We believe that the language is now acceptable for the review process.

**Comment 5:** Given the high number of tables and figures, it is recommended that detailed captions be provided for tables and figures. The captions should provide a clear and detailed explanation of the content or representation of each table or figure, thus facilitating the reader's understanding of the data presented.

**Author’s response:** Thank the reviewer for the comment. We have made the relevant revisions as requested. **(See Lines 505-509;586-587;)**.

**Comment 6:** Please ensure that every table and figure is appropriately referenced within the manuscript.

**Author’s response:** Thank the reviewer for the comment.

**Author’s response:** Thank the reviewer for the comment. We have revised the fields that did not use the relevant table contents.

We have reviewed point by point in the new revised version, marked by red character, according to your comments. I hope the above answers and revisions are satisfactory, and the paper will be acceptable for publication in ***PLOS ONE***.

Best wishes,

Dr. Gui-mei Zhang
